# Comparative Histology of the Cornea and Palisades of Vogt in Various Non-Human Primates

**DOI:** 10.3390/vetsci13010109

**Published:** 2026-01-22

**Authors:** Joanna Klećkowska-Nawrot, Aleksander Chrószcz, Abit Aktaş, Wojciech Paszta, Karolina Goździewska-Harłajczuk, Dominik Poradowski

**Affiliations:** 1 Division of Animal Anatomy, Department of Biostructure and Animal Physiology, Faculty of Veterinary Medicine, Wrocław University of Environmental and Life Sciences, Kożuchowska 1, 51-631 Wrocław, Poland; 2 Department of Histology and Embryology, Faculty of Veterinary Medicine, Istanbul University-Cerrahpasa, 34320 Istanbul, Türkiye; 3 Wrocław Zoological Garden, Z. Wróblewskiego 1-5, 51-618 Wrocław, Poland

**Keywords:** cornea, limbal region, non-human primates, palisades of Vogt

## Abstract

The cornea is essential for proper ocular function, yet its histological structure varies considerably among animal species. Of particular importance are the palisades of Vogt in the limbal region, as they serve as a niche for limbal epithelial stem cells involved in corneal epithelial regeneration. This study was conducted on 73 eyeballs collected from 18 species of non-human primates originating from the Wrocław Zoological Garden (Poland). Eyeballs were fixed, processed, and embedded in paraffin. Four-micrometer sections were stained with Mayer’s H&E and PAS. Microscopically, the cornea showed either a four-layered pattern (anterior corneal epithelium, corneal stroma, Descemet’s membrane, posterior corneal epithelium) or a five-layered pattern when Bowman’s layer was present. A four-layered cornea occurred in the ring-tailed lemur, gray mouse lemur, Guianan squirrel monkey, Angolan colobus, and L’Hoest’s monkey, while the remaining species showed a five-layered structure with Bowman’s layer. The anterior corneal epithelium varied between species in thickness and number of cell layers (central region: 2–3 to 10–15 layers; 11.81 ± 0.43 µm to 44.23 ± 0.69 µm; peripheral region: 4–5 to 9–11 layers; 8.63 ± 2.57 µm to 42.45 ± 8.61 µm). Bowman’s layer ranged from 1.18 ± 0.01 µm to 3.22 ± 0.05 µm. The corneal stroma thickness differed markedly (237.96 ± 9.64 µm to 1438.29 ± 16.38 µm), as did Descemet’s membrane (4.92 ± 0.20 µm to 43.45 ± 0.49 µm), along with PAS reaction intensity. In the limbus, palisades of Vogt ranged from weakly to clearly developed; well-defined crypt-like structures were observed in the red-bellied lemur, red ruffed lemur, black-and-white ruffed lemur, Guianan squirrel monkey, L’Hoest’s monkey, Celebes crested macaque, and yellow baboon. The limbal epithelium also varied in thickness (5–6 to 15–17 cell layers). These results confirm distinct species-specific differences in corneal and limbal morphology that may reflect ecological conditions and functional adaptation. The presented data provides a comparative reference for veterinary ophthalmology and for studies on corneal epithelial regeneration involving limbal stem cells.

## Simple Summary

The cornea is the most external and transparent structure of the eye influenced directly by the life conditions existing in the external environment. It plays an important role both in eye defense and light reception by the sense of vision. The study describes the microstructure of the cornea in non-human primates. The number of analyzed structures was 73, coming from 18 animal species. The histological staining visualized the subsequent structures of the cornea and allowed for the structural identification and description. The differences observed in the cornea architecture, such as the thickness of the corneal epithelium, the presence of corneal membranes and corneal stroma, together with the variability of the corneal limbus morphology, proved the importance of both environmental and phylogenetic factors in the architecture of the studied organ. Animal adaptation to the lifestyle in a specific environment seems to be crucial for the explanation of differences between species. The achieved results can be important for better understanding of the eye’s morphology, physiology and pathology, which is helpful for further studies.

## 1. Introduction

The cornea is a key structure required for normal ocular function, serving as the main refractive element of the eye and a transparent protective barrier for intraocular tissues [1]. Together with the sclera, it forms the fibrous layer of the eyeball [1,2,3]. In humans, it is a thin, convex, transparent tissue located at the front of the globe [4,5,6]. In the human, the cornea is composed of several layers with distinct roles. The cornea is avascular; its outer surface is formed by the anterior corneal epithelium, which is maintained by the tear film, is densely innervated, and regenerates rapidly [7]. Beneath it lies Bowman’s layer, a collagenous membrane contributing to resistance to trauma and deformation [7]. The proper corneal stroma (proper substance of the cornea; commonly referred to as the corneal stroma) provides most of the cornea’s strength and contributes critically to its transparency [1,4]. A thin pre-Descemet layer (often referred to as Dua’s layer) was proposed as a distinct anatomical entity in the human cornea in 2013 [8]; however, its status as an independent corneal layer remains debated [9,10,11]. To date, it has been described primarily in the human cornea [8,12], with analogous observations reported ex vivo in the canine cornea [13].

Therefore, in the present study we refer to it as a term used in the human literature and do not assume its presence as a separate layer in non-human primates. Descemet’s membrane separates the corneal stroma from the posterior corneal epithelium and acts as a resilient protective barrier. The posterior corneal epithelium (i.e., the corneal endothelium) consists of hexagonal cells responsible for maintaining corneal dehydration and preventing aqueous humor from entering the cornea [4,14,15,16]. In this manuscript, the nomenclature of corneal layers follows Nomina Histologica Veterinaria [17] and Nomina Anatomica Veterinaria [1]. The limbus of the cornea is the transitional zone between the cornea and the sclera and is adjacent to the bulbar conjunctiva [18,19,20,21,22,23]. It contains conjunctival folds (palisades of Vogt) with vascular networks and niches for limbal epithelial stem cells (LESCs) [23]. This microenvironment supports LESC maintenance and provides factors needed for their survival [22,24,25]. LESCs replenish the corneal epithelium and contribute to immune homeostasis, wound healing, and regeneration; in the healthy human eye, the anterior epithelium is renewed every 3–10 days [22,24,25]. When LESC function is impaired, epithelial renewal is compromised, which may lead to loss of transparency and structural integrity of the cornea [24]. Limbal stem cell deficiency can be congenital or acquired (e.g., burns, long-term contact lens wear, infections, or neoplasms). Current management includes pharmacotherapy, surgical approaches, and autologous limbal transplantation, while allogenic transplantation may be required in total deficiency [22,26,27,28,29]. Because corneal structure and physiology vary across species, comparative studies are important, especially since animal corneal tissue is commonly used in translational research and therapeutic development [3,30,31]. However, available histological data in non-human primates are often limited to single species or selected parameters, and comprehensive cross-family comparisons—particularly regarding the limbal region and palisades of Vogt—remain scarce [3,30,31].

In addition, non-human primates develop corneal and limbal disorders (e.g., keratitis, corneal dystrophies, or limbal epithelial stem cell deficiency), and understanding species-specific microanatomy supports accurate diagnosis and treatment in veterinary ophthalmology [3,30,31]. In this context, engineered equivalents of human corneal endothelium have been evaluated for function in monkey models [32].

This study focuses on histological comparisons of the cornea and palisades of Vogt across various non-human primate families. The primary objective is to identify the microstructural differences and similarities in these ocular regions between different primate groups. The significance of these structures in veterinary ophthalmology is highlighted, as understanding their histology is essential for diagnosing and treating ocular diseases in nonhuman primates. Variations in the corneal layers and palisades of Vogt may affect susceptibility to conditions such as keratitis, corneal dystrophies, and limbal epithelial stem cell deficiency. Recognizing these differences is crucial for advancing veterinary ophthalmology and improving eye care in non-human primates.

## 2. Materials and Methods

### 2.1. Collection of Specimens

The material for this study was collected from 73 non-human primates from Lemuridae, Cheirogaleidae, Callitrichidae, Cebidae, Atelidae, and Cercopithecidae, representing 18 species coming from the Wrocław Zoological Garden (Poland). The species included the Alaotra reed lemur (*Hapalemur alaotrensis*), red-bellied lemur (*Eulemur rubriventer*), crowned lemur (*Eulemur coronatus*), ring-tailed lemur (*Lemur catta*), red ruffed lemur (*Varecia rubra*), black-and-white ruffed lemur (*Varecia variegata*), gray mouse lemur (*Microcebus murinus*), common marmoset (*Callithrix jacchus*), golden-headed lion tamarin (*Leontopithecus chrysomelas*), Guianan squirrel monkey (*Saimiri sciureus*), black-and-gold howler (*Alouatta caraya*), Angolan colobus (*Colobus angolensis*), L’Hoest’s monkey (*Allochrocebus lhoesti*), putty-nosed monkey (*Cercopithecus cephus*), red-capped mangabey (*Cercocebus torquatus*), Celebes crested macaque (*Macaca nigra*), rhesus macaque (*Macaca mulatta*), and yellow baboon (*Papio cynocephalus*). Information on systematics, number of individuals, collection dates, IUCN status, and age at death (years) (from Wrocław Zoological Garden records) is presented in Table 1 [33,34,35,36,37,38,39,40,41,42,43,44,45]. Review of the available medical and veterinary records revealed no systemic conditions likely to affect the ocular surface or tear production; routine pre-mortem ophthalmic examinations were not performed. Post-mortem examination showed no gross injuries or pathological changes in the head or globe. None of the non-human primates were euthanized for the purpose of this study; all specimens were obtained *post-mortem*. These animals were collected between 2013 and 2025, and the samples are currently stored in the Division of Animal Anatomy, Department of Biostructure and Animal Physiology at Wrocław University of Environmental and Life Sciences (Poland). 

### 2.2. Ethical Statement

According to Polish and European law, research on tissues obtained *post-mortem* does not require approval from an Ethics Committee. This is in accordance with the Journal of Laws of the Republic of Poland (Act of 15 January 2015, on the Protection of Animals Used for Scientific or Educational Purposes) and European law (Directive 2010/63/EU of the European Parliament and of the Council of 22 September 2010, on the protection of animals used for scientific purposes). Written permits for the collection of *post-mortem* animal material were obtained from the District Veterinary Officer in Wrocław, Poland. The permits were issued under the following reference numbers and names: No. PIW Wroc. UT-45/5/16 (dr. Joanna Klećkowska-Nawrot) and No. PIW Wroc. UT-45/6/16 (dr. Karolina Goździewska-Harajczuk).

### 2.3. Histological and Histochemical Staining (H&E, PAS) and Semi-Quantitative Scoring

Due to the extremely rare nature of the material obtained from non-human primates, the histological and histochemical analysis was conducted over an extended period as specimens became available from all individuals. Immediately after the deaths of the animals, the eyeballs were removed and placed in 4% buffered formaldehyde (Chempur, Piekary Śląskie, Poland) for at least 72 h, followed by rinsing in running water for 24 h. They were then processed in a vacuum tissue processor (ETP RVG3, Intelsint, Villarbasse, Italy) and embedded in paraffin (Chempur, Piekary Śląskie, Poland). The samples were cut into 4 µm sections using a Slide 2003 sliding microtome (Pfm A.g., Cologne, Germany). Mayer’s hematoxylin (Sigma-Aldrich, Taufkirchen, Germany) and eosin (Sigma-Aldrich, Taufkirchen, Germany) and periodic acid–Schiff (PAS) (Sigma-Aldrich, Taufkirchen, Germany) staining methods were applied [46]. The PAS stain was used to visualize Descemet’s membrane because this structure is rich in glycoproteins and proteoglycans, which produce a strongly positive reaction to the presence of carbohydrates. The positive staining result also enabled the assessment of its integrity, thickness, and potential pathological changes, which is essential for analyzing the condition of the endothelium and extracellular matrix. The resulting slides were examined using a Zeiss Axio Scope A1 light microscope (Carl Zeiss, Jena, Germany) and evaluated using a scoring system based on a previously described standard protocol [46]. The results were interpreted as follows: (−) indicates a negative reaction, (−/+) and (+) indicate a weak positive reaction, (++) and (++/+++) indicate a moderate positive reaction, and (+++) indicates a strong positive reaction. For the histological description of the structures examined, *Nomina Anatomica Veterinaria* [1] and *Nomina Histologica Veterinaria* [14] were used. Histometric measurements (the values of six randomly selected corneal structures: anterior corneal epithelium, anterior limiting membrane, proper substance of cornea, and posterior limiting membrane) were statistically processed (mean ± standard deviation) StatisticaPL 13.0 program (StatSoft, Kraków, Poland) and were performed using Axio Vision Rel. 4.8 software (Carl Zeiss, Jena, Germany). Relative (percentage) contributions of corneal layers were calculated for the central cornea as: (layer thickness/sum of measured layer thicknesses) × 100, using the central anterior epithelial thickness and the thicknesses of Bowman’s layer (if present), proper substance of cornea, and Descemet’s membrane. The endothelium was not included because it was not measured.

## 3. Results

**Figure 1:**
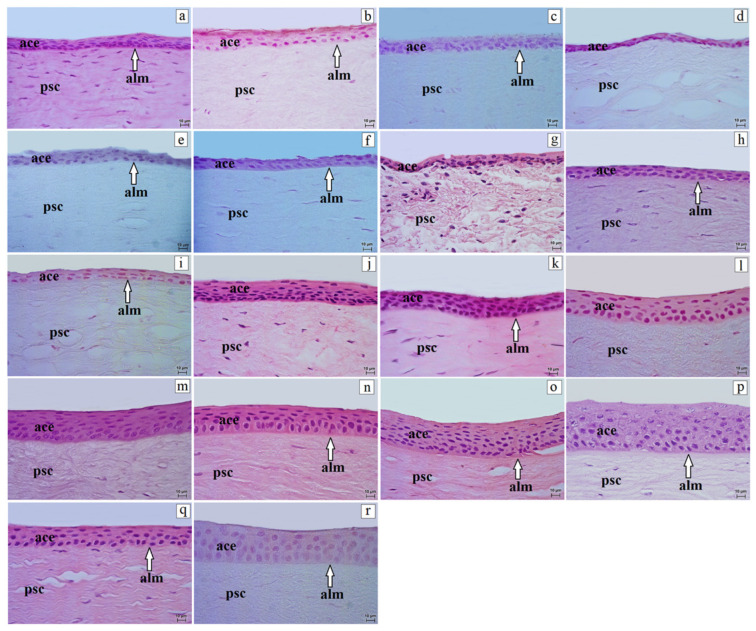
Histology of the anterior corneal epithelium in the central cornea and Bowman’s layer in non-human primates. (**a**)—Alaotra reed lemur, (**b**)—red-bellied lemur, (**c**)—crowned lemur, (**d**)—ring-tailed lemur, (**e**)—red ruffed lemur, (**f**)—black-and-white ruffed lemur, (**g**)—gray mouse lemur, (**h**)—common marmoset, (**i**)—golden-headed lion tamarin, (**j**)—Guianan squirrel monkey, (**k**)—black-and-gold howler, (**l**)—Angolan colobus, (**m**)—L’Hoest’s monkey, (**n**)—putty-nosed monkey, (**o**)—red-capped mangabey, (**p**)—Celebes crested macaque, (**q**)—rhesus macaque, (**r**)—yellow baboon. ace—anterior corneal epithelium; alm—anterior limiting membrane (Bowman’s layer; white arrow); psc—proper substance of cornea. H&E stain. Scale bar: (**a**–**r**) = 10 µm.

Results are reported for the entire cohort of non-human primates. Age at death (years) is provided in Table 1 and was not used as a grouping variable in the analyses. The cornea in several non-human primate species, including the ring-tailed lemur, gray mouse lemur, Guianan squirrel monkey, Angolan colobus, and L’Hoest’s monkey consisted of four layers: anterior corneal epithelium, proper substance of the cornea, posterior limiting membrane (Descemet’s membrane), and posterior corneal epithelium (endothelium). In the remaining examined species, the cornea consisted of five layers because the anterior limiting membrane (Bowman’s layer), located between the anterior corneal epithelium and the proper substance of the cornea, was present (Figure 1).

The anterior corneal epithelium was a stratified non-keratinized squamous epithelium composed of the following cell layers: superficial cells, intermediate cells, and basal cells. As in stratified squamous epithelia, the classification “squamous” refers to the superficial cell layer, whereas basal cells may be cuboidal/low columnar. The superficial cells were polygonal with a flattened nucleus and clear cytoplasm, and many cells showed features of desquamation. The intermediate layers consisted of wing-like cells. In epithelia composed of only 2–3 cell layers (e.g., the ring-tailed lemur), an intermediate (wing-cell) layer was not distinguishable as a separate tier and therefore only basal and superficial layers were identified. Large and round nuclei were observed in the common marmoset, black-and-gold howler, rhesus macaque, and yellow baboon, whereas flattened nuclei were noted in the remaining species. Basal cells were predominantly cuboidal (isoprismatic) to low columnar (cylindrical), with an oval, large nucleus (Figure 1). The anterior corneal epithelium showed a variable number of cell layers in the central region. The lowest number occurred in the ring-tailed lemur (2–3), golden-headed lion tamarin (4–5), crowned lemur (4–6), black-and-white ruffed lemur, and Alaotra reed lemur (4–6), and the putty-nosed monkey (4–7), whereas the highest number was found in the red-capped mangabey (10–15) and Celebes crested macaque (8–17) (Table 2). Variation was also observed in the peripheral epithelium, with the fewest layers in the red ruffed lemur and ring-tailed lemur (4–5), and the most in the common marmoset (9–10), Celebes crested macaque (7–10), and Angolan colobus (7–11) (Table 2). The thickness of the anterior corneal epithelium differed between the central and peripheral regions. The thinnest central epithelium occurred in the ring-tailed lemur (11.81 ± 0.43 µm) and Alaotra reed lemur (12.91 ± 0.68 µm), whereas the thickest was found in the Celebes crested macaque (44.23 ± 0.69 µm) and yellow baboon (43.73 ± 0.65 µm). Similarly, in the peripheral region, the thinnest epithelium was noted in the ring-tailed lemur (8.63 ± 2.57 µm), and the thickest in the Celebes crested macaque (42.45 ± 8.61 µm) (Table 2).

The Bowman’s layer was observed in the Alaotra reed lemur, red-bellied lemur, crowned lemur, red ruffed lemur, black-and-white ruffed lemur, common marmoset, golden-headed lion tamarin, black-and-gold howler, putty-nosed monkey, red-capped mangabey, Celebes crested macaque, rhesus macaque, and yellow baboon. Among these species, the thinnest Bowman’s layer occurred in the black-and-white ruffed lemur (1.18 ± 0.01 µm), whereas the thickest was present in the yellow baboon (3.22 ± 0.05 µm) (Table 3). The Bowman’s layer was composed of thin collagen fibers.

**Figure 2:**
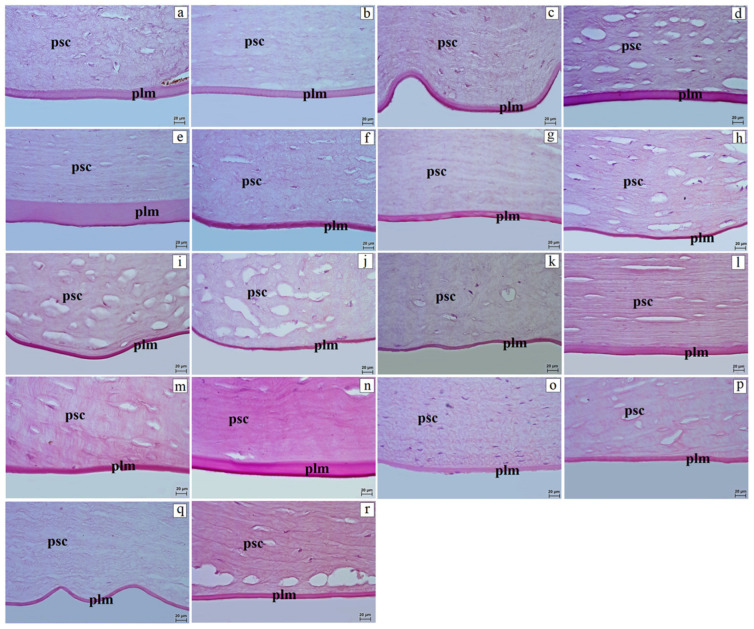
Histology of Descemet’s membrane in non-human primates. (**a**)—Alaotra reed lemur, (**b**)—red-bellied lemur, (**c**)—crowned lemur, (**d**)—ring-tailed lemur, (**e**)—red ruffed lemur, (**f**)—black-and-white ruffed lemur, (**g**)—gray mouse lemur, (**h**)—common marmoset, (**i**)—golden-headed lion tamarin, (**j**)—Guianan squirrel monkey, (**k**)—black-and-gold howler, (**l**)—Angolan colobus, (**m**)—L’Hoest’s monkey, (**n**)—putty-nosed monkey, (**o**)—red-capped mangabey, (**p**)—Celebes crested macaque, (**q**)—rhesus macaque, (**r**)—yellow baboon. plm—posterior limiting membrane (Descemet’s membrane); psc—proper substance of the cornea. PAS stain. Scale bar: (**a**–**r**) = 10 µm.

The proper substance of the cornea consisted of a uniform collagen fibril matrix. Between collagen lamellae, layers of flattened and elongated corneal stromal fibroblasts were present (Figure 1 and Figure 2). In the Alaotra reed lemur, gray mouse lemur, common marmoset, Guianan squirrel monkey, black-and-gold howler, Celebes crested macaque, and rhesus macaque, the proper substance of the cornea contained very few corneal stromal fibroblasts. The corneal stroma was the thickest layer of the cornea and varied among species. The thinnest stroma occurred in the Angolan colobus (237.96 ± 9.64 µm) and Alaotra reed lemur (317.32 ± 4.07 µm), whereas the thickest was found in the Guianan squirrel monkey (1438.29 ± 16.38 µm) (Table 3).

The posterior limiting membrane also showed variable thickness. The thinnest Descemet’s membrane occurred in the common marmoset and Guianan squirrel monkey (4.92 ± 0.20 to 5.84 ± 0.11 µm), whereas the thickest was observed in the red ruffed lemur (43.45 ± 0.49 µm) (Figure 2, Table 3). Histochemical analysis using PAS staining revealed that the strongest reaction (+++) occurred in the golden-headed lion tamarin, ring-tailed lemur, and putty-nosed monkey; a moderate reaction (++) was noted in the crowned lemur, black-and-white ruffed lemur, common marmoset, gray mouse lemur, L’Hoest’s monkey, rhesus macaque, and yellow baboon; while a weak positive reaction (+) occurred in the remaining species (Figure 2). The posterior epithelium of the cornea was lined by a single-layer squamous epithelium (endothelium). When expressed as proportional thickness, the proper substance of the cornea constituted the dominant component of the measured corneal thickness in all species (typically >90%), whereas the anterior epithelium accounted for a minor fraction. Bowman’s layer, when present, contributed <0.5% of the measured thickness, while Descemet’s membrane generally accounted for ~0.4–6.0% (Table 4).

**Figure 3:**
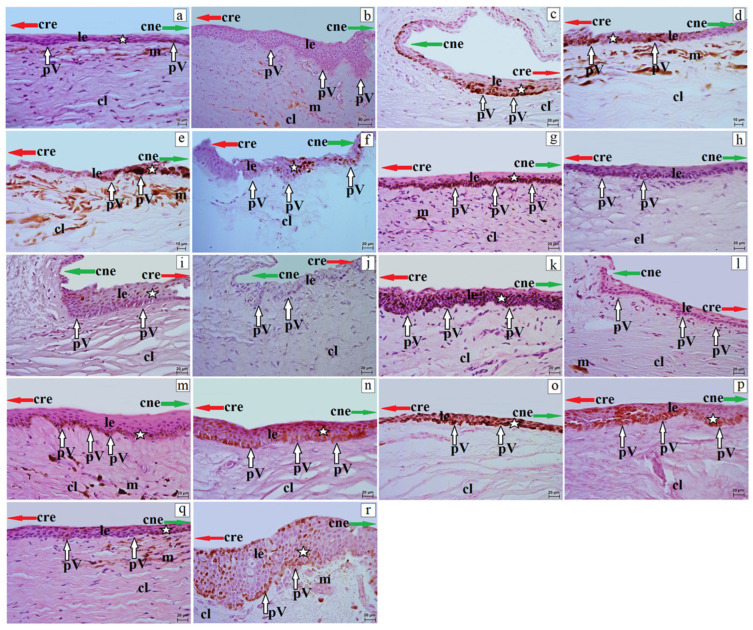
Histology of the Palisades of Vogt in non-human primates. (**a**)—Alaotra reed lemur, (**b**)—red-bellied lemur, (**c**)—crowned lemur, (**d**)—ring-tailed lemur, (**e**)—red ruffed lemur, (**f**)—black-and-white ruffed lemur, (**g**)—gray mouse lemur, (**h**)—common marmoset, (**i**)—golden-headed lion tamarin, (**j**)—Guianan squirrel monkey, (**k**)—black-and-gold howler, (**l**)—Angolan colobus, (**m**)—L’Hoest’s monkey, (**n**)—putty-nosed monkey, (**o**)—red-capped mangabey, (**p**)—Celebes crested macaque, (**q**)—rhesus macaque, (**r**)—yellow baboon. cl—corneal limbus; cre—corneal epithelium (red arrow); cne—conjunctival epithelium (green arrow); le—limbal epithelium; m—melanocytes; pV—palisades of Vogt (white arrows); white asterisk—presence of melanocytes in the limbal epithelium. H&E stain. Scale bars: (**b**) = 50 µm; (**c**,**f**–**r**) = 20 µm; (**a**,**d**,**e**) = 10 µm.

The corneal limbus was located at the junction of the cornea and sclera. It was characterized by the loss of the anterior limiting membrane (in species in which Bowman’s layer was present) and by the organization of collagen fibers with clusters of pigment cells in the red-bellied lemur, ring-tailed lemur, red ruffed lemur, gray mouse lemur, Angolan colobus, L’Hoest’s monkey, rhesus macaque, and yellow baboon (Figure 3).

At the border between the corneal and conjunctival epithelia, palisades of Vogt were observed. In the red-bellied lemur, red ruffed lemur, black-and-white ruffed lemur, Guianan squirrel monkey, L’Hoest’s monkey, Celebes crested macaque, and yellow baboon, the palisades of Vogt were clearly developed as crypt-like structures, whereas in the remaining species these formations were moderately or poorly defined (Figure 3). The limbal epithelium consisted of a variable number of cell layers (Table 5). The lowest number of layers occurred in the ring-tailed lemur and red-capped mangabey (5–6), gray mouse lemur (5–7), and common marmoset (5–8), whereas the highest number was found in the yellow baboon (15–17) and Celebes crested macaque (15–16) (Table 5).

The superficial layer consisted of flattened cells with squamous nuclei; the intermediate layer consisted of wing cells with oval nuclei; and the basal layer consisted of cylindrical cells (red-bellied lemur, crowned lemur, black-and-white ruffed lemur, gray mouse lemur, common marmoset, golden-headed lion tamarin, Angolan colobus, L’Hoest’s monkey, putty-nosed monkey) or isoprismatic cells (Alaotra reed lemur, ring-tailed lemur, red ruffed lemur, Guianan squirrel monkey, black-and-gold howler, red-capped mangabey, rhesus macaque, Celebes crested macaque, and yellow baboon) (Figure 3). Except for the red-bellied lemur, common marmoset, Guianan squirrel monkey, and Angolan colobus (in which melanocytes were absent in all epithelial layers), the limbal epithelium showed a variable number of melanocytes located in different epithelial layers (Table 5, Figure 3).

## 4. Discussion

The cornea is a transparent structure located on the anterior surface of the eye, covering the iris, pupil, and anterior chamber [47]. It serves multiple roles: optical—accounting for approximately 65–75% of the eye’s total refractive power; protective—acting as a mechanical barrier safeguarding deeper ocular structures against trauma and infection; filtering—absorbing harmful UV radiation; nutritive—enabling the transport of oxygen and nutrients to deeper layers of the eye; and immunological—contributing to defense mechanisms against pathogens [48,49]. Any disruption to the cornea’s function or structural integrity (e.g., scarring, ulcers, inflammation, or dystrophies) can impair transparency and visual acuity and, in severe cases, may necessitate corneal transplantation [50]. The cornea’s regenerative capacity depends on the type and depth of injury: superficial damage typically heals well through regeneration of the anterior corneal epithelium, whereas deeper lesions may lead to permanent scarring [51,52]. Summarizing, the corneal morphology influences its physiological function, and any pathological changes are able to cause severe eye disorders.

A limitation of this study is that age at death varied within and across species and was not modeled as a covariate; therefore, age-related effects on the measured parameters cannot be excluded. Nevertheless, although the general histological structure of the cornea in nonhuman primates is similar, we observed significant morphometric differences in corneal thickness, epithelial layer number, and anterior epithelial organization. These variations may reflect adaptations to environmental conditions and lifestyle (e.g., diurnal versus nocturnal activity, or terrestrial versus arboreal habitats). As reported by Kirk [53], visual adaptations in primates, including corneal size, are strongly linked to ecological lifestyle. In nocturnal species such as the gray mouse lemur, a relatively large corneal diameter in proportion to the eye facilitates greater light absorption [54]. In contrast, diurnal species, which are more exposed to UV radiation, often present a thicker anterior epithelium or a more developed Bowman’s layer [55]. In arboreal species, where environmental conditions require accurate near vision (e.g., navigating branches and foliage), a more developed ciliary muscle and a more elastic lens may enhance accommodation and visual acuity [56,57,58,59]. A recent study by Olopade et al. [60] in tree-dwelling squirrels indicated that corneal thickness and structure are closely tied to habitat, with UV exposure and environmental irritants contributing to corneal thickening. Above-mentioned morphological variations were also observed in our histological analysis. The differences in corneal layer number, thickness, and epithelial structure were observed among non-human primate species and may reflect adaptive pressure related to activity patterns (diurnal/nocturnal), habitat (arboreal/terrestrial), and environmental exposure (e.g., dust, humidity, pathogens). For instance, in gray mouse lemur, the cornea consisted of only four layers. It lacked Bowman’s layer, which may reflect adaptation to reduced mechanical stress and UV exposure typical of nocturnal and arboreal environments. In such cases, simplified corneal architecture may be advantageous under low-light conditions, where light transmission is more important than external protection. Interestingly, the absence of Bowman’s layer was also noted in ring-tailed lemur, Guianan squirrel monkey, Angolan colobus, and L’Hoest’s monkey, despite their diurnal and partially terrestrial lifestyles [61,62,63]. This suggests that the presence or absence of this layer may not be strictly dictated by circadian activity or habitat type, but instead may reflect phylogenetic factors or other adaptations, such as differences in the stroma, pre-corneal tear film composition, or epithelial characteristics. In contrast, Bowman’s layer was present in 13 other species similar to capuchin monkey (*Sapajus* sp.) [64] and western lowland gorilla (*Gorilla gorilla gorilla*) [65], which may point to a need for enhanced structural reinforcement of the cornea in response to increased exposure to mechanical or chemical irritants. Although the Bowman’s layer was thin (1.18–3.22 µm), it may still play an essential role in stabilizing the corneal surface and serving as a barrier against trauma or infection [66]. The quantitative and qualitative variability was also found in the corneal epithelia. Notable interspecies differences were also observed in the anterior corneal epithelium. Ring-tailed lemur and Alaotra reed lemur exhibited the thinnest central epithelial layers (11.81–12.91 µm), while the thickest epithelium (>43 µm) was noted in Celebes crested macaque and yellow baboon. A thicker epithelium may offer better protection against UV radiation, particulate matter, and mechanical trauma. The highest number of epithelial cell layers (15–17) was observed in red-capped mangabey and Celebes crested macaque, suggesting a strong barrier function. Variability in the nuclear morphology of wing-like and basal cells (e.g., round in common marmoset, black-and-gold howler, rhesus macaque, yellow baboon, and flattened in others) may indicate species-specific differences in epithelial regeneration and micro-injury response.

We also found considerable variation in stromal thickness and corneal stromal fibrocytes density. The thickest stroma (>1400 µm) was observed in Guianan squirrel monkey, and the thinnest in Angolan colobus and Alaotra reed lemur. Low corneal stromal fibrocytes density in some species (e.g., rhesus macaque and Guianan squirrel monkey) may affect transparency and regenerative capacity. Descemet’s membrane thickness also varied significantly—from <6 µm in common marmoset to >43 µm in red ruffed lemur. Such differences may influence endothelial function and corneal resilience to intraocular pressure changes or the risk of anterior segment pathologies [67]. Observed differences in anterior epithelial thickness, epithelial cell layer count, presence or absence of Bowman’s layer, and the appearance of the palisades of Vogt may represent species-specific protective adaptations to differing light exposure, mechanical resistance requirements, and ecological niches. To facilitate interspecies comparisons, we expressed the thickness of individual corneal layers as a percentage of the measured total corneal thickness, because proportional values are often more informative for interspecies comparisons than absolute micrometer measurements alone. This approach helps to reduce the effect of overall scaling and biological variability in corneal thickness, which is known to be tightly regulated and can change with factors such as age and body-size scaling [68]. Importantly, human data consistently show that the stroma constitutes the dominant component of corneal thickness (close to ~90%), whereas the epithelium represents a much smaller fraction, and Bowman’s layer and Descemet’s membrane contribute only minimally [69,70,71]. In our non-human primates, the same general pattern was observed (stromal dominance), but the proportional analysis highlighted species-related differences in how the measured thickness is distributed across layers, which may be less obvious when comparing absolute values only. For clarity, these percentages were calculated relative to the sum of the layers measured in the central cornea (epithelium + Bowman’s layer, if present + stroma + Descemet’s membrane), with endothelium not included because it was not measured.

The corneal limbus also showed variation in the prominence of the palisades of Vogt and in the number of limbal epithelial layers. Red-capped mangabey, Celebes crested macaque and yellow baboon exhibited particularly well-developed palisades on routine light microscopy, whereas in other species they were only moderately or poorly demarcated. Because our assessment is primarily morphological and qualitative, these observations should be interpreted as differences in the morphological appearance of the limbal region rather than as direct evidence of functional differences in regenerative capacity. The palisades are considered to represent the niche of limbal epithelial stem cells (LESCs), which support continuous renewal of the corneal surface [22,68]. However, confirming species-specific differences at the cellular or ultrastructural level would require additional approaches, such as immunohistochemical characterization of stem cell–associated markers and/or ultrastructural analysis by electron microscopy.

Dysfunction of the LESCs or their niche can result in corneal opacity, neovascularization, conjunctivalization, and vision loss, as documented in several clinical and experimental studies [72,73]. Our findings indicate that while the corneal structure is broadly similar across primates, interspecies anatomical differences observed by routine histology—including Bowman’s layer, epithelial thickness, and the morphological appearance of the limbal region—may be relevant when selecting appropriate non-human primate models for corneal research. Species such as the rhesus macaque and yellow baboon, which exhibited a well-developed Bowman’s layer and a relatively thick anterior epithelium, and in which palisades of Vogt appeared more prominent, may represent closer morphological analogs to the human cornea than lemurs. Nevertheless, functional extrapolations should be made cautiously and ideally supported by targeted marker-based studies [74,75,76,77,78].

Since the samples were collected *post-mortem* from a zoological facility, our study is subject to certain limitations. Although the animals had access to outdoor enclosures, and no ocular pathologies were documented in veterinary records during life or after death, a functional in vivo assessment of the cornea was not feasible due to the *post-mortem* nature of the study, and ethical and technical constraints related to non-clinical anesthesia. All individuals were adults that died from various causes (natural death, illness, trauma), providing a degree of homogeneity in terms of age and maintenance conditions, but limiting direct comparison with data from free-ranging primates. Moreover, environmental conditions in the zoological facility—including artificial lighting, climate control, humidity, and pathogen exposure—may have influenced corneal histology and should be considered when interpreting our results in the context of wild populations.

## 5. Conclusions

Our study shows that non-human primates show clear, species-specific differences in the structure of the cornea and limbus. These differences mainly include the presence or absence of Bowman’s layer, as well as noticeable variation in the thickness of the anterior corneal epithelium and in the number of its cell layers—even among closely related species. Differences were also observed in other corneal components. Across species, the proper substance of cornea represented the major proportion of the measured central corneal thickness (83.5–97.7%; median ~95.5%), whereas the anterior epithelium contributed 1.46–12.0% (median ~2.6%), Descemet’s membrane 0.40–6.04% (median ~1.15%), and Bowman’s layer—when present—0–0.46% (median ~0.19%). In the limbal region, palisades of Vogt were present in all examined species, but their appearance varied from weakly defined to clearly developed, and in some species they formed crypt-like structures. The observed differences may result from both evolutionary adaptation and environmental influences. The presented findings may serve as a useful reference in veterinary ophthalmology and in comparative research on the maintenance and regeneration of the corneal epithelium. Because these observations are based on routine histology and qualitative morphological assessment, further studies using specific cellular markers and/or ultrastructural methods are warranted to better characterize interspecies variation in the limbal stem cell niche.

## Figures and Tables

**Table 1:** Taxonomic and conservation status of the examined non-human primates from Wrocław Zoological Garden (Poland).

Suborder	Infraorder	Parvorder	Superfamily	Family	Subfamily	Tribe/ Group	Species	Age at Death (Years), Range (min-max)	Activity Pattern/ Arboreality Level	Red List of iucn (2025-2)	Number of Collection Animals /Year of Collection
Strepsirrhini	Lemuriformes	–	Lemuroidea	Lemuridae	Hapalemurinae	–	Alaotra reed lemur *Hapalemur alaotrensis*	<1–7	Diurnal/ Arboreal (reedbeds, marsh vegetation)	CR decreasing	3 /2018, 2019
					Lemurinae	–	red-bellied lemur *Eulemur rubriventer*	3–17	Cathemeral/ Arboreal	VU decreasing	4 /2018, 2019, 2021
						–	crowned lemur *Eulemur coronatus*	2	Cathemeral, mostly diurnal/ Arboreal	EN decreasing	1 /2020
						–	ring-tailed lemur *Lemur catta*	<1–7	Diurnal/ Semi-terrestrial (most terrestrial among lemurs)	EN decreasing	10 /2021, 2022, 2025
						–	red ruffed lemur *Varecia rubra*	<1–20	Diurnal/ Highly arboreal	CR decreasing	4 /2019, 2020, 2022
						–	black-and-white ruffed lemur *Varecia variegata*	<1–29	Diurnal/ Highly arboreal	CR decreasing	5 /2018, 2019, 2021, 2022
		–	Cheirogaleoidea	Cheirogaleidae	Cheirogaleinae	–	gray mouse lemur *Microcebus murinus*	9	Nocturnal/ Arboreal	LC decreasing	1 /2019
Haplorhini	Simiiformes	Platyrrhini	Ceboidea	Callitrichidae	Callitrichinae	–	common marmoset *Callithrix jacchus*	13	Diurnal/ Arboreal	LC decreasing	1 /2021
						–	golden-headed lion tamarin *Leontopithecus chrysomelas*	15	Diurnal/ Arboreal	EN decreasing	1 /2021
				Cebidae	Saimiriinae	Saimiriini	Guianan squirrel monkey *Saimiri sciureus*	<1–7	Diurnal/ Arboreal	LC decreasing	9 /2017, 2018, 2019, 2021
				Atelidae	Alouattinae	Alouattini	black-and-gold howler *Alouatta caraya*	<1–22	Diurnal/ Arboreal, semi-terrestrial	NT decreasing	9 /2016, 2018, 2019, 2021, 2022, 2025
		Catarrhini	Cercopithecoidea	Cercopithecidae	Colobine	Colobini	Angolan colobus *Colobus angolensis*	12–18	Diurnal/ Arboreal	VU decreasing	4 /2021, 2025
					Cercopithecinae	Cercopithecini	L’Hoest’s monkey *Allochrocebus lhoesti*	12–18	Diurnal/ Semi-terrestrial	VU decreasing	2 /2021, 2025
							putty-nosed monkey *Cercopithecus nictitans*	16	Diurnal/ Arboreal	NT decreasing	1 /2018
							red-capped mangabey *Cercocebus torquatus*	<1–15	Diurnal/ Semi-terrestrial	EN decreasing	4 /2017, 2018, 2023
						Papionini	Celebes crested macaque *Macaca nigra*	7	Diurnal/ Terrestrial, semi-arboreal	CR decreasing	3 /2017, 2018, 2019
							rhesus macaque *Macaca mulatta*	26	Diurnal/ Semi-arboreal	LC	1 /2018
							yellow baboon *Papio cynocephalus*	N/A	Diurnal/ Terrestrial	LC stable	10 /2013, 2017, 2018, 2019

N/A—non-applicable.

**Table 2:** Morphometric features of the anterior corneal epithelium in non-human primates.

Species	Family	Anterior Corneal Epithelium									
		Number of Cellular Layers (Central)	Number of Superficial Cell Layers	Number of Intermediate Cell Layers	Number of Basal Cell Layers	Thickness (Central) (µm)	Number of Cellular Layers (Peripheral)	Number of Superficial Cell Layers	Number of Intermediate Cell Layers	Number of Basal Cell Layers	Thickness (Peripheral) (µm)
Alaotra reed lemur *Hapalemur alaotrensis*	Lemuridae	4–6	3	3–4	1–2	12.91 (±0.68)	4–6	2–3	1	1–2	11.01 (±2.54)
red-bellied lemur *Eulemur rubriventer*		5–8	1–2	3	1–2	20.41 (±0.46)	6–8	2–3	3	1–2	13.79 (±4.73)
crowned lemur *Eulemur coronatus*		4–6	1–2	2–3	1	18.19 (±0.37)	7–8	1–2	4–5	1–2	14.71 (±3.71)
ring-tailed lemur *Lemur catta*		2–3	1	1–2	1	11.81 (±0.43)	4–5	1–2	2	1	8.63 (±2.57)
red ruffed lemur *Varecia rubra*		5–9	1–3	2–4	2	19.98 (±0.51)	4–5	1–2	3	1	15.14 (±4.27)
black-and-white ruffed lemur *Varecia variegata*		4–6	1–2	1–3	1	16.69 (±0.95)	5–7	1–2	2–4	1	14.08 (±3.28)
grey mouse lemur *Microcebus murinus*	Cheirogaleidae	5–7	1–2	3–4	1	14.48 (±0.65)	6–8	1–3	2–4	1	11.29 (±3.02)
common marmoset *Callithrix jacchus*	Callitrichidae	7–10	1–2	5–6	1–2	16.08 (±0.48)	9–10	1–2	5–8	1–2	12.41 (±3.39)
golden-headed lion tamarin *Leontopithecus chrysomelas*		4–5	1–2	1–3	1	16.09 (±0.63)	6–8	1–2	2–4	1–2	13.59 (±3.21)
Guianan squirrel monkey *Saimiri sciureus*	Cebidae	8–11	2–3	4–6	1–2	27.31 (±0.59)	5–6	1–3	2	1	23.39 (±5.39)
black-and-gold howler *Alouatta caraya*	Atelidae	6–12	1–4	2–6	1–2	25.54 (±0.73)	5–6	2	2–3	1	20.69 (±5.17)
Angolan colobus *Colobus angolensis*	Cercopithecidae	7–12	1–4	5–6	1–2	34.18 (±1.25)	7–11	1–3	5–6	1–2	32.18 (±6.56)
L’Hoest’s monkey *Allochrocebus lhoesti*		9–14	1–3	5–9	1–2	38.18 (±0.47)	5–9	1–2	3–4	1–3	34.41 (±7.45)
putty-nosed monkey *Cercopithecus nictitans*		4–7	1–3	2–3	1–2	33.84 (±0.7)	5–8	1–2	3–4	1–2	26.67 (±7.01)
red-capped mangabey *Cercocebus torquatus*		10–15	1–2	1–2	1–3	21.89 (±0.61)	5–6	1–3	2–3	1	17.93 (±4.41)
Celebes crested macaque *Macaca nigra*		8–17	1–4	6–10	1–3	44.23 (±0.69)	7–10	2–3	4–5	1–2	42.45 (±8.61)
rhesus macaque *Macaca mulatta*		5–10	1–3	3–4	1–3	23.14 (±0.8)	7–9	1–3	3–4	1–2	17.16 (±4.95)
yellow baboon *Papio cynocephalus*		5–9	1–3	3–4	1–2	43.73 (±0.65)	6–8	1–3	3–4	1	33.89 (±9.13)

**Table 3:** Thickness (µm) of Bowman’s layer, proper substance of the cornea, and Descemet’s membrane in non-human primates.

Species	Family	Bowman’s Layer	Proper Substance of the Cornea	Descemet’s Membrane
Alaotra reed lemur *Hapalemur alaotrensis*	Lemuridae	1.32 (±0.02)	317.32 (±4.07)	18.21 (±0.15)
red-bellied lemur *Eulemur rubriventer*		1.47 (±0.03)	742.87 (±3.92)	15.04 (±0.2)
crowned lemur *Eulemur coronatus*		1.32 (±0.02)	814.73 (±4.34)	9.79 (±0.36)
ring-tailed lemur *Lemur catta*		absent	780.76 (±2.74)	16.66 (±0.43)
red ruffed lemur *Varecia rubra*		1.31 (±0.01)	654.53 (±14.71)	43.45 (±0.49)
black-and-white ruffed lemur *Varecia variegata*		1.18 (±0.01)	631.01 (±7.44)	12.2 (±0.12)
grey mouse lemur *Microcebus murinus*	Cheirogaleidae	absent	409.84 (±1.43)	11.91 (±0.11)
common marmoset *Callithrix jacchus*	Callitrichidae	2.85 (±0.09)	591.51 (±4.12)	4.92 (±0.2)
golden-headed lion tamarin *Leontopithecus chrysomelas*		1.58 (±0.03)	757.59 (±3.1)	6.85 (±0.07)
Guianan squirrel monkey *Saimiri sciureus*	Cebidae	absent	1438.29 (±16.38)	5.84 (±0.11)
black-and-gold howler *Alouatta caraya*	Atelidae	2.61 (±0.04)	1057.63 (±22.41)	6.28 (±0.04)
Angolan colobus *Colobus angolensis*	Cercopithecidae	absent	237.96 (±9.64)	12.89 (±0.14)
L’Hoest’s monkey *Allochrocebus lhoesti*		absent	930.85 (±8.77)	11.21 (±0.06)
putty-nosed monkey *Cercopithecus nictitans*		3.01 (±0.06)	876.05 (±9.18)	20.66 (±0.29)
red-capped mangabey *Cercocebus torquatus*		3.14 (±0.04)	839.03 (±2.86)	8.89 (±0.01)
Celebes crested macaque *Macaca nigra*		1.89 (±0.04)	979.15 (±4.18)	8.88 (±0.02)
rhesus macaque *Macaca mulatta*		2.95 (±0.12)	1038.88 (±7.9)	6.31 (±0.02)
yellow baboon *Papio cynocephalus*		3.22 (±0.05)	1159.48 (±5.2)	7.31 (±0.03)

**Table 4:** Relative (%) contribution of measured corneal layers (central cornea). Percentages were calculated as: layer thickness/(sum of measured layer thicknesses) × 100, using central anterior epithelial thickness (Table 2) and Bowman’s layer (if present), proper substance of the cornea, and Descemet’s membrane thicknesses (Table 3). Layers marked as absent were treated as 0.

Species	Family	Anterior Corneal Epithelium (%)	Bowman’s Layer (%)	Proper Substance of the Cornea (%)	Descemet’s Membrane (%)
Alaotra reed lemur *Hapalemur alaotrensis*	Lemuridae	3.69	0.38	90.73	5.21
red-bellied lemur *Eulemur rubriventer*		2.62	0.19	95.27	1.93
crowned lemur *Eulemur coronatus*		2.16	0.16	96.53	1.16
ring-tailed lemur *Lemur catta*		1.46	0.00	96.48	2.06
red ruffed lemur *Varecia rubra*		2.78	0.18	91.00	6.04
black-and-white ruffed lemur *Varecia variegata*		2.52	0.18	95.45	1.85
grey mouse lemur *Microcebus murinus*	Cheirogaleidae	3.32	0.00	93.95	2.73
common marmoset *Callithrix jacchus*	Callitrichidae	2.61	0.46	96.12	0.80
golden-headed lion tamarin *Leontopithecus chrysomelas*		2.06	0.20	96.86	0.88
Guianan squirrel monkey *Saimiri sciureus*	Cebidae	1.86	0.00	97.75	0.40
black-and-gold howler *Alouatta caraya*	Atelidae	2.34	0.24	96.85	0.58
Angolan colobus *Colobus angolensis*	Cercopithecidae	11.99	0.00	83.49	4.52
L’Hoest’s monkey *Allochrocebus lhoesti*		3.89	0.00	94.96	1.14
putty-nosed monkey *Cercopithecus nictitans*		3.62	0.32	93.84	2.21
red-capped mangabey *Cercocebus torquatus*		2.51	0.36	96.11	1.02
Celebes crested macaque *Macaca nigra*		4.28	0.18	94.68	0.86
rhesus macaque *Macaca mulatta*		2.16	0.28	96.98	0.59
yellow baboon *Papio cynocephalus*		3.60	0.27	95.53	0.60

**Table 5:** Morphological characteristics of the limbus epithelium (palisades of Vogt) in non-human primates.

Species	Family	Limbus Epithelium				
		Total Number of Cell Layers	Number of Superficial Cell Layers	Number of Intermediate Cell Layers	Number of Basal Cell Layers	Presence of Melanocytes
Alaotra reed lemur *Hapalemur alaotrensis*	Lemuridae	6–8	1–2	2–3	1–2	+ (all cell layers)
red-bellied lemur *Eulemur rubriventer*		14–15	1–3	10–12	1–2	absent
crowned lemur *Eulemur coronatus*		6–7	1–3	3	1	+++ (intermediate and basal cell layers)
ring-tailed lemur *Lemur catta*		5–6	2	2–3	1	+++ (all cell layers)
red ruffed lemur *Varecia rubra*		5–6	1	3–4	1–2	+++ (all cell layers)
black-and-white ruffed lemur *Varecia variegata*		8–14	1–3	4–7	3–4	++ (all cell layers)
grey mouse lemur *Microcebus murinus*	Cheirogaleidae	5–7	1–2	3–4	1	+/++ (intermediate and basal cell layers)
common marmoset *Callithrix jacchus*	Callitrichidae	5–8	1–2	3–4	1–2	absent
golden-headed lion tamarin *Leontopithecus chrysomelas*		13–15	1–3	9–10	1–3	+/++ (peripheral and intermediate cell layers)
Guianan squirrel monkey *Saimiri sciureus*	Cebidae	5–8	1–3	3	1–2	absent
black-and-gold howler *Alouatta caraya*	Atelidae	11–13	1–2	9	1–2	+ (intermediate cell layers)
Angolan colobus *Colobus angolensis*	Cercopithecidae	8–11	1–2	6–7	1–2	absent
L’Hoest’s monkey *Allochrocebus lhoesti*		8–13	1–3	6–8	1–2	+ (basal cell layers)
putty-nosed monkey *Cercopithecus nictitans*		9–13	1–2	7–9	1–2	+/++ (all cell layers)
red-capped mangabey *Cercocebus torquatus*		5–6	1–2	3–4	1	+++ (all cell layers)
Celebes crested macaque *Macaca nigra*		15–16	2–3	10–12	1–2	+/++ (intermediate and basal cell layers)
rhesus macaque *Macaca mulatta*		10–12	1–3	5–8	1–2	+ (all cell layers)
yellow baboon *Papio cynocephalus*		15–17	1–2	12–13	1–2	++/+++ (all cell layers)

## Data Availability

The raw data supporting the conclusions of this article will be made available by the authors on request.
